# Axillary lymph node dissection for breast cancer utilizing Harmonic Focus^®^

**DOI:** 10.1186/1477-7819-9-90

**Published:** 2011-08-15

**Authors:** Katherine T Ostapoff, David Euhus, Xian-Jin Xie, Madhu Rao, Amy Moldrem, Roshni Rao

**Affiliations:** 1 Department of Surgery, Division of Surgical Oncology, University of Texas Southwestern Medical Center, 5323 Harry Hines Blvd., Dallas, TX, USA 75390-9155; 2 Department of Clinical Sciences, University of Texas Southwestern Medical Center, 5323 Harry Hines Blvd., Dallas, TX, USA 75390-9155

## Abstract

**Background:**

For patients with axillary lymph node metastases from breast cancer, performance of a complete axillary lymph node dissection (ALND) is the standard approach. Due to the rich lymphatic network in the axilla, it is necessary to carefully dissect and identify all lymphatic channels. Traditionally, these lymphatics are sealed with titanium clips or individually sutured. Recently, the Harmonic Focus^®^, a hand-held ultrasonic dissector, allows lymphatics to be sealed without the utilization of clips or ties. We hypothesize that ALND performed with the Harmonic Focus^® ^will decrease operative time and reduce post-operative complications.

**Methods:**

Retrospective review identified all patients who underwent ALND at a teaching hospital between January of 2005 and December of 2009. Patient demographics, presenting pathology, treatment course, operative time, days to drain removal, and surgical complications were recorded. Comparisons were made to a selected control group of patients who underwent similar surgical procedures along with an ALND performed utilizing hemostatic clips and electrocautery. A total of 41 patients were included in this study.

**Results:**

Operative time was not improved with the use of ultrasonic dissection, however, there was a decrease in the total number of days that closed suction drainage was required, although this was not statistically significant. Complication rates were similar between the two groups.

**Conclusion:**

In this case-matched retrospective review, there were fewer required days of closed suction drainage when ALND was performed with ultrasonic dissection versus clips and electrocautery.

## Background

Complete axillary staging is a critical component of the management of advanced breast cancer. Currently, for patients with no clinical evidence of axillary metastases, this information is obtained via sentinel node biopsy[1]. However, for patients with pathologically confirmed axillary metastases via positive sentinel node biopsy or percutaneous axillary biopsy complete axillary lymph node dissection (ALND) should be considered [2,3]. Although the recent results of ACOSOG Z0011 trial suggest that patients with T1/T2 tumors undergoing breast conserving therapy do not require completion axillary dissection, patients with more advanced lesions, patients undergoing total mastectomy and patients with palpable nodes continue to require ALND for staging and to reduce the risk of axillary recurrence [4]. Complete axillary node dissection, although necessary, continues to carry the risks of complications such as seroma, chronic lymphatic leaks and lymphedema.

The axilla is filled with a rich lymphatic network that requires careful dissection to identify all channels. Traditionally, ALND has been performed using titanium clips and suture ligation along with bovie electrocautery[5]. Inadequate sealing of lymphatics can result in lymphatic leaks, infection, lymphedema and seromas[6]. The frequency of these complications can vary from 3-85% for seroma formation and 5-49% for lymphedema[6-12].

Recently, ultrasonic devices have been developed which allow for precise simultaneous cutting and hemostasis with minimal damage to surrounding tissues. It functions by denaturing collagen and elastin in tissue bundles, vessel walls, and lymphatics to form a coagulum[5]. Ultrasonic dissection provides the ability to seal vascular and lymphatic conduits with limited collateral damage.

The Harmonic Focus^® ^is a handheld ultrasonic dissection device which allows lymphatics to be sealed without utilization of clips or ties. It has been available since October 2007 and is designed for open procedures. It has been used for dissection in surgery of the liver, gallbladder, thyroid and breast [13-18]. It has been used in other lymphatic dissections in an attempt to reduce lymphatically related complications and has been shown to reduce operative times and reduce lymphatic spillage with similar rates of lymph node harvest for both modified lateral neck dissections and central neck dissections in thyroid cancer[16,17]. The ultrasonic dissector has also been shown to reduce blood loss[12].

We hypothesize that ALND performed with Harmonic Focus^® ^will decrease operative time and post-operative complications. Here we present our pilot study investigating the role of Harmonic Focus^® ^as method to reduce axillary complications.

## Methods

All women at the University of Texas Southwestern Medical Center undergoing ALND for confirmed breast cancer metastasis from January 1, 2005 to December 1, 2009 were eligible. Nearly 250 ALND's were performed during this time period. In 2007, ultrasonic dissection became the standard technique for ALND for 3 fellowship trained breast surgeons (RR, AM, DE) at this large, high volume, academic medical center. Twenty eight patients underwent ALND with the Harmonic^® ^scalpel. Patient records were reviewed after IRB approval. These patients were then matched for number and type of procedures performed at the time of ALND with thirteen historical case controls by procedure performed by the same surgeons. All patients had pathologically confirmed axillary metastasis prior to ALND by either sentinel node biopsy or percutaneous axillary biopsy. Additionally, none of the patients in either group had breast reconstruction at the time of axillary dissection. Due to the surgeon preference for utilizing ultrasonic dissection once it became available, one to one matched pair analysis was not possible, however, the thirteen controls reviewed were performed by the same surgeons that performed the ultrasonic dissections to minimize surgeon specific variables.

### Surgery

Skin incisions were made with a scalpel. If concurrent breast surgery occurred at the time of ALND, mastectomy was performed using either tumescent technique or bovie electrocautery. For axillary lymphadenectomy, all tissue inferior to the axillary vein, between the anterior border of the latissimus dorsi and medial to the border of the pectoralis minor was removed, thus including all level 1 and 2 nodes. The axilla was closed with 3.0 Vicryl interrupted suture and the skin was re-approximated with 4.0 Monocryl. At the time of surgery, 1 closed suction 19 french Jackson-Pratt drain was placed for patients with partial mastectomy and axillary dissection and 2 closed suction drains were placed for modified radical mastectomy patients. Drains were then removed on an outpatient basis when output was less than 30 cc/day for 2 consecutive days. Operative time was obtained by review of the anesthesia record and operating room nurse record. Patients were discharged routinely on postoperative day one with drains in place and self recorded the output after teaching.

Incidence of ipsilateral lymphedema, seroma and wound complications was noted upon review of follow-up appointments in any department (Surgery/Radiation Oncology, Medical Oncology or Emergency department). Categorical variables were analyzed using χ^2 ^test, continuous variables were analyzed using ANOVA testing using SPSS 12.0. All endpoint analyses were analyzed using paired t-test. Multiple comparisons were not adjusted for.

## Results

Over the 2 year period, 28 patients underwent axillary dissection using Harmonic scalpel^®^. Median follow-up time for both groups was 2.0 years. Patients in both groups had similar demographics as well as pathology and exposure to chemotherapy (Table 1). Additionally, there was no statistically significant difference in T stage between the control and ultrasonic dissection group (p = 0.222). Patients were matched to controls undergoing similar procedures by the same surgeons (Table 2).

**Table 1:** Patient Demographics

	Control n = 13	Harmonic n = 28	P value
Age at Diagnosis	49.4	52	P = 0.415

BMI	25	31.1	P = 0.090

Ethnicity			P = 0.234

White	43% (n = 5)	21% (n = 6)	

Black	21% (n = 3)	42% (n = 12)	

Hispanic	29% (n = 4)	25% (n = 7)	

Asian	0	11% (n = 3)	

Other	7% (n = 1)	0	

Tumor Size	3.4	3.91	P = 0.545

Clinical T Stage			P = 0.222

T1	N = 6	N = 6	

T2	N = 2	N = 11	

T3	N = 4	N = 6	

T4	N = 1	N = 5	

Histology			P = 0.378

Invasive ductal	11 (79%)	24 (86%)	

Invasive lobular	1 (14%)	2 (7%)	

Other	1 (7%)	0	

Combination	0	2 (7%)	

Type of Surgery			P = 0.566

PM	5	10	

TM	8	18	

Neoadjuvant chemotherapy	43% (n = 6)	32% (n = 9)	P = 0.614

Adjuvant chemotherapy	78% (n = 11)	79% (n = 22)	P = 0.601

BMI = body mass index PM = partial mastectomy, TM = total mastectomy

**Table 2:** Paired Surgical Procedures

	Control (13)	Harmonic (28)
ALND alone	3	3

ALND +1 minor procedure *	1	5

ALND+PM+mediport	3	2

ALND+PM+SLND+mediport	1	3

MRM	1	7

MRM+ mediport	1	1

TM+SLNB+ALND	1	2

ALND+TM+ mediport+ contralateral SLNB+contralateral TM	2	5

*minor procedure included sentinel node biopsy, partial mastectomy, or mediport placement

ALND = axillary lymph node dissection, PM = partial mastectomy, SLNB = sentinel node biopsy, MRM = modified radical mastectomy, TM = total mastectomy

There were no differences in operative time, postoperative day 1 output or overall complications between the groups. There was a difference in days to drain removal (15.9 vs 12.4 days p = 0.07), however, this was not statistically significant. There was no difference in rates of seromas, wound complications or lymphedema between the two groups (Table 3).

**Table 3:** Endpoint Analysis

	Control n = 13	Harmonic n = 28	P value
Operative Time *	208.5 min	209 min	P = 0.965

Output POD#1 *	134 cc	134.4 cc	P = 0.982

Days JP remained*	**15. 9 days **	**12. 4 days **	**P = 0.07 **

Complications	4 (31%)	10 (36%)	P = 0.788

Seroma	2 (15.4%)	2 (7%)	P = 0.377

Wound infection	1 (8%)	4 (13%)	P = 0.486

Lymphedema	0 (0%)	4 (13%)	P = 0.170

* = paired t test

## Discussion

Given the significant morbidity of ALND and the costs associated with their management, multiple studies have tried to identify risks factors as well as methods to reduce complications. Risk factors for seroma formation include MRM rather than PM[9], use of electrocautery for ALND[19], older age, patient weight, BMI[20] and drainage output at 48 hours (>50 cc/day)[21]. The role of axillary drainage has been a particular area of focus. Drains have been shown to reduce seroma rates compared to no drainage at all[21-23]. Lower overall drain volume[24], days to drain removal[25,26] and drainage less than 30-50 cc/day[27] prior to removal have all been shown to reduce seroma formation.

Given the observed residual dead space after ALND, attempts have been made to use hemostatic agents to reduce seroma formation. Use of thrombin spray failed to show any significant reduction in seroma formation or days required for closed suction drainage[22]. In a prospective randomized trial, the use of fibrin glue did not prevent or reduce seroma formation, aspiration volume, or complications[28]. Similarly, Berger found no difference in axillary drainage time, seroma formation, drain volume or local inflammation following use of a fibrin glue coated collagen patch[29]. Use of argon beam decreased operative blood loss, but not seroma formation[30].

Variances in surgical technique have also been explored. Classe JM et al attempted to reduce hospital stays by using axillary padding and 3 layered closure of the axilla rather than closed suction drainage, there were no differences in post-operative seroma rates (p = 0.9) or needle aspiration (p = 0.94)[31]. Wound closure by suturing skin flaps to the axillary space resulted in longer operative times, but did decrease the number of days until drain removal, as well as overall drain volume thereby resulted in reduced rates of seroma formation[32]. Additionally, several studies have evaluated the feasibility of axillary reverse mapping (ARM) a technique in which one can intraoperatively map the axilla [33,34] In a small pilot study this was shown to prevent lymphedema[35]. Another newer technique is LYMPHA, which entails a lymphatic to venous anastomosis at the time of axillary dissection [36]. Lymphedema (as defined as increase in arm volume at serial follow-up measurements) in this study, at 18 month follow-up, was defined by a reduction in arm circumference. After ALND with LYMPHA, only 4.4% of patients developed lymphedema, versus 30% in patients who underwent ALND alone [36].

Investigators have also attempted to use ultrasonic dissectors (Ligasure^® ^device) in a similar fashion to that presented here. Galatius et al found no differences in seroma incidence using the ultrasonic dissector, however, overall seroma rate was 66% and all drains were removed on post operative day 5 regardless of output[37]. Lumachi et al found there was no reduction in seroma rate using this method but did find on univariate analysis that risk factors for seroma formation included BMI, tumor size, number of involved nodes, total drainage before drain removal and time to drain removal[38].

This is the first investigation in an American institution, evaluating the use of the Harmonic Focus^® ^to reduce complications for ALND. Adwani et al found decreased blood loss but no difference in days until drain removal, seroma formation or number of treatments for seromas, and were thus unable to show improved outcomes in England[18]. An Indian study similarly found reduced blood loss but also found reduced drainage volume and drainage days but found no difference in seroma formation or operative time (seroma rate 16% vs 22%)[39]. Manouras et al in Greece found no complications with the use of the Harmonic^® ^scalpel for ALND in 60 patients at 3 years of follow-up[40]. Although impressive, only 22% of these patients had positive nodes, a factor which has been shown to increase the risk for axillary complications. Additionally, comparisons may be difficult as the majority of these patients had lower stage tumors (5% T3) than the present study[41].

We found fewer days to drain removal with the use of ultrasonic dissection. Although there was no difference in operative time, seroma formation, wound complications or lymphedema, reduced drainage time has been shown to reduce complication rates[20]. Larger studies with increased statistical power may be able to detect other differences. Many of our patients had bilateral procedures done at the time of ALND with and without immediate reconstruction, which increases expected operative times and increases the risk of complications.

The Harmonic^® ^scalpel, in addition to reducing the days of drain placement, can also potentially reduce healthcare costs. Fewer days of drainage can reduce the number of clinic visits and potentially the need for additional studies. Also, there is a potential for reduced costs of equipment given that the current institutional cost of an automatic clip applier is $185, with the use of 2-3 per case and the Harmonic Focus^® ^is approximately $400.

By allowing faster removal of closed suction drainage after ALND, Harmonic focus^® ^may allow a more rapid transition to the initiation of systemic therapy as suggested by our pilot study.

## Conclusions

In this retrospective, pilot study, the use of ultrasonic dissection resulted in trends suggesting decreased days of closed suction drainage when compared to traditional techniques utilizing clips and electrocautery. Ultrasonic dissection was not associated with decreased operative time or lower rates of post-operative complications. Further studies with larger sample sizes are required to confirm these results.

## Abbreviations

ALND: axillary lymph node dissection; PM: partial mastectomy; TM: total mastectomy; SLNB: sentinel lymph node biopsy; BMI: body mass index.

## Competing interests

The authors declare that they have no competing interests.

## Authors' contributions

KO: study concept and design, data acquisition, analysis and interpretation of data, drafting of manuscript, critical revision, DE: critical revision, patients, XX: analysis and interpretation of data, critical revision, MR: cost analysis, critical revision, AM: critical revision, patients RR: study concept and design, data acquisition, patients, analysis and interpretation of data, drafting of manuscript, critical revision. The authors have reviewed this manuscript and agree with its contents in its final form.
